# A prospective observational study to evaluate the effect of social and personality factors on continuous positive airway pressure (CPAP) compliance in obstructive sleep apnoea syndrome

**DOI:** 10.1186/s12890-017-0393-7

**Published:** 2017-03-22

**Authors:** Atul Gulati, Masood Ali, Mike Davies, Tim Quinnell, Ian Smith

**Affiliations:** 1 Consultant respiratory medicine, New Cross Hospital, Heath Road, Wolverhampton, West Midlands WV10 0QP UK; 2 Consultant respiratory medicine, Southend University Hospital, Prittlewell Chase, Westcliffe on Sea, Essex SS0 0RY UK; 3 Consultant respiratory medicine, Respiratory Support and Sleep Centre, Papworth Hospital, Papworth Everard, Cambridge, CB23 3RE UK

**Keywords:** Obstructive sleep apnoea, CPAP compliance, Socio-economic status, Unemployed, Depression, Type D personality, Physician’s prediction

## Abstract

**Background:**

Compliance with CPAP treatment for OSAS is not reliably predicted by the severity of symptoms or physiological variables. We examined a range of factors which could be measured before CPAP initiation to look for predictors of compliance.

**Methods:**

This was a prospective cohort-study of CPAP treatment for OSAS, recording; socio-economic status, education, type D personality and clinician’s prediction of compliance.

**Results:**

We recruited 265 subjects, of whom 221 were still using CPAP at 6 months; median age 53 years, M: F, 3.4:1, ESS 15 and pre-treatment ODI 21/h. Median compliance at 6 months was 5.6 (3.4– 7.1) hours/night with 73.3% of subjects using CPAP ≥4 h/night. No association was found between compliance and different socio-economic classes for people in work, type D personality, education level, sex, age, baseline ESS or ODI. The clinician’s initial impression could separate groups of good and poor compliers but had little predictive value for individual patients. Compared to subjects who were working, those who were long term unemployed had a lower CPAP usage and were more likely to use CPAP < 4 h a night (OR 4.6; *p* value 0.011). A high Beck Depression Index and self-reported anxiety also predicted poor compliance.

**Conclusions:**

In our practice there is no significant association between CPAP compliance with socio-economic status, education or personality type. Long term unemployed or depressed individuals may need more intensive support to gain the optimal benefit from CPAP.

## Background

Obstructive sleep apnoea syndrome (OSAS) is a common condition, which can lead to low health status [1] through sleepiness, poor concentration and marital discord [2]. It increases the likelihood of accidents including road traffic collisions [3] and is probably an independent risk factor for cardiovascular mortality and morbidity [4, 5]. Continuous positive airway pressure (CPAP) treatment can reverse daytime sleepiness and in case controlled series is associated with a fall in the number of car crashes and the incidence of strokes and heart attacks [6–8]. The benefit on a number of symptom measures is proportional to the amount of time spent using CPAP [9]. In some series that reported a reduction in cardiovascular disease with CPAP the comparator group with worse outcomes comprised non-acceptors of CPAP [10]. Compliance with CPAP is therefore key to its effectiveness. Initial acceptance of treatment has been reported to be as good as 84% [11] but historical series have reported that as few as 46% of initial acceptors subsequently used CPAP for more than 4 h/night [12]. In more recent studies of interventions to improve compliance the use of CPAP has been found to range between 2 to 8 h [13]. Given the large number of patients treated with CPAP a significant number of symptomatic OSA patients are still left with suboptimal usage.

The initial pattern of compliance is one of the better predictors of long-term use [14]. Interventions that are directed at increasing compliance may be costly [15]. For both of these reasons, it would be most helpful if clinicians could identify the patients who are likely to fail to comply well with treatment before it is initiated, to focus interventions at the start of treatment in those most likely to need extra support. There have been studies investigating associations with poor compliance. Several have reported the impact of nasal and pressure related side effects, but these only become apparent after a period of treatment. The severity of OSA has been shown to have predictive value in some [16] but not all studies [17]. Recent reports have shown associations for both low socio-economic group [18–21] and type D personality (the tendency to suppress emotional distress) [22] with poor compliance though the latter was retrospective. The physician’s ability to predict which patients will be good and poor compliers with CPAP has not been extensively investigated. More recent published work supported adopting a common sense patient centred approach, assessing medical, technical and behavioural factors in improving CPAP compliance [23].

We conducted this prospective study in newly diagnosed OSAS patients to determine which socio-economic and personality factors identified before the initiation of CPAP might be associated with lower rates of initial acceptance and sub-optimal long-term compliance with the therapy.

This study was approved by the Cambridgeshire 1 research and ethics committee (08/H0304/72).

## Methods

All individuals referred to a specialist respiratory support and sleep centre and seen initially in our outpatient clinic with suspected OSAS were considered for the study. The diagnosis of OSAS was established on the basis of interview with a sleep specialist and overnight oximetry showing an ODI ≥ 10 per hour. Multi-channel respiratory sleep studies or polysomnography (PSG) were performed if patients with a history strongly suggestive of OSAS had an ODI < 10 per hour or patients had an ODI > 10 per hour and a cardio-respiratory or neurological condition which might produce nocturnal hypoxemia or cause central apnoea or Cheyne - Stokes respiration.

Patients who had a diagnosis of OSAS confirmed and were considered for CPAP treatment were approached to participate in the study. After informed consent subjects completed a range of questionnaires as described below. The following factors were examined looking for their association with CPAP compliance:

### Primary factors

- Socio-economic status – derived by national statistics socio-economic classification (NS-SEC), self coded version [24]. The self-coded version of the national statistics socioeconomic classification was used to assess SEC. This scale takes into account; an individual’s occupational and employment status, size of the employing organisation, self-employed vs. employee, managerial vs. no supervisory role and allocates individuals to one of 6 classes (one class being long term unemployed).
- Type D personality – also known as distressed personality, reflects an individual’s ability to suppress emotions. This was assessed using a validated tool (DS-14 scale) comprising 7 questions related to negative affectivity or social inhibition [25].

### Secondary factors

- The physician’s estimate of the likelihood that the patient would accept CPAP treatment and subsequently comply with the treatment.
- Depression – assessed by Beck’s depression inventory 2 (BDI-2). An existing diagnosis of depression was noted. All subjects completed a BDI-2 questionnaire. Those with a score < 13 were classified as normal, 14–19 mild depression, 20–28 moderate depression, and 29–63 severe depression. It is noted that this tool is designed to assess the severity of depression and is not intended as a diagnostic tool
- Anxiety - self report of previous diagnosis.
- The patient’s sex
- Level of education- Educational history was categorised as no formal qualification, school certificate, higher school certificate or bachelor degree.
- Sleep specific quality of life scores (sleep apnoea quality of life index-SAQLI) score.
- Epworth sleepiness scale score (ESS)
- Severity of OSA as assessed by 4% oxygen desaturation index (ODI).

The physician who assessed the patient in clinic estimated the likelihood of the patient accepting the treatment and continuing to use CPAP for > 4 h/night, expressed as a percentage on a visual analogue scale (from 0 to 100). The estimates were based on their impression after reviewing the patient for the first time in clinic and assessing the initial response of patients to the offer of CPAP treatment. The investigators did not ask the clinicians to consider any particular information when evaluating the likelihood of acceptance and compliance with CPAP. The physicians marking the visual analogue scale had from 2 to 25 years of experience in managing sleep disorders.

After consenting to participate in the study, patients were admitted for overnight manual titration with CPAP. Treatment was commenced at a single centre and standard local titration protocols were followed. Next day, patients were discharged on a fixed pressure CPAP device, with the pressure having been determined from the titration night. Choice of mask (nasal vs. full face vs. total face) and humidification usage was influenced by patient choice and comfort, rather than unselective use of either. All the patients had access to the sleep physiologist for a mask or humidification review if needed. This approach potentially reduced the selection bias and was more in keeping with real life clinical management of OSAHS with CPAP.

During subsequent follow-up at 6–10 weeks and 6 months; SAQLI and BDI-2 scores were repeated. Additional questions were asked to assess the individual’s experience of CPAP treatment.

### Outcome measures

Individuals who used CPAP and were still using it at second follow-up appointments up to 6 months were designated acceptors. CPAP compliance was measured using an inbuilt counter in the CPAP machine, during the follow-up visits at 6–10 weeks and 6 months. Average CPAP use of less than 4 h/night was considered sub-optimal compliance.

### Statistical analysis

Using the socio-economic status variable, it was estimated that 240 patients would give a predictive power to detect a one hour difference in compliance between the different groups. When the results were analysed most of the variables were found to be non-normally distributed and groups were compared using the non-parametric Mann–Whitney U test. To assess the ability of the physician’s confidence that the subject would use CPAP to predict compliance, receiver operator characteristic (ROC) analysis was performed.

## Results

The study recruited over a period of 7 months. In this period 566 patients were initiated on CPAP therapy in the sleep centre, 410 of whom were first seen in clinic and were therefore available for the study. Of these, 265 consented to participate in the study. There were 205 men (male to female ratio 3.4:1). Expressed as medians (IQR) the age of participants was 53.0 (44–61) years, ESS 15.0 [11–17], BMI 34.8 (31.6- 41.7) kg/m^2^, ODI 21.0 (12.3- 41.2) per hour. Type D personality was found in 120 (45%) subjects. At baseline 31.7% were on antidepressants or said that they had previously been diagnosed with depression by their general practitioner and 44.6% of subjects had a BDI-2 score of > 13.

**Fig. 1:**
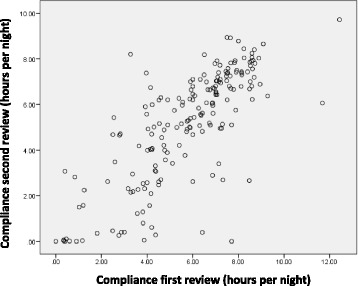
Correlation between early (6–10 weeks) and late CPAP compliance (6 months)

From the cohort of 265 subjects, 9 were switched to a bi-level device, 5 were excluded (one was diagnosed with an upper airway malignancy, another was cured of OSA by tonsillectomy, 3 did not attend any follow-up visit during the study period), 5 withdrew consent. The initial acceptance was calculated as the proportion of individuals still using CPAP after 6 months. Two hundred and twenty one study subjects (89.8%) were still using CPAP at 6 months. There was a strong correlation between the early compliance measured as hours of use at first follow-up, (6–10 weeks) and 6 months compliance with CPAP (*r* = 0.77) (Fig. 1). The median use at first follow up and 6 months were, 6.0 (4.2-7.5) and 5.6 (3.4- 7.1) hours/night respectively. By our definition of < 4 h/night, 19.0% were using CPAP sub-optimally at first visit and 26.7% at 6 months.

**Fig. 2:**
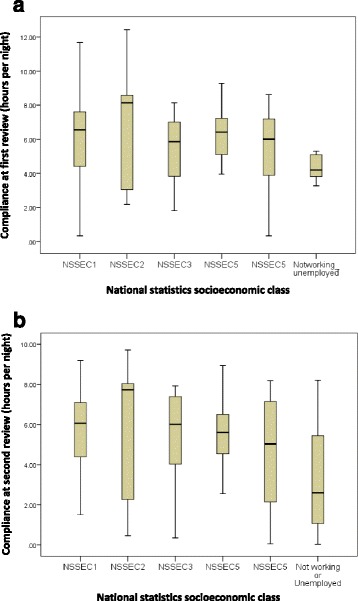
Hours of CPAP use in different socioeconomic class. **a** Socioeconomic class vs. CPAP compliance at first visit. **b** Socioeconomic class vs. CPAP compliance at 6 month

The distribution of different SEC categories within the study population is shown in Table 1. The hours of use in each SEC category at first follow up and at 6 months is shown in Fig. 2a and b respectively. Compared to individuals who were working (or retired from work), those who were long term unemployed had lower median hours of CPAP usage at first follow up and at 6 months (Table 2). This group was also more likely to use CPAP sub-optimally (<4 h/night) at first follow up visit and at 6 months (OR 3.3, and OR 4.6 respectively, see Table 3).

**Table 1:** Distribution between different socio-economic status groups for study population with OSAS

Socio-economic status	Number(percent)
1	107 (40.4%)
2	8 (3.0%)
3	45 (17.0%)
4	47 (17.7%)
5	24 (9.1%)
Long term unemployed- not working	19 (7.2%)
Missing	15 (5.7%)

**Table 2:** Median hours of use for subjects using CPAP in different sub groups defined by study characteristics

	First follow-up (6–10 weeks)		6 month follow-up	
	Median hours of use (IQR) per night	*P* value	Median hours of use (IQR) per night	*P* value
Long term unemployed vs. people in work (or retired)	4.2 (3.8–5.3) vs. 6.3 (4.3– 7.5)	*0.028*	2.61 (1.0 – 5.7) vs. 5.96 (4.0– 7.2)	*0.016*
Education : Bachelor degree vs. less, formal education	6.04 (4.14–7.49) vs. 5.90 (4.53–7.42)	*0.61*	5.75 (3.4–7.13) vs. 6.02 (5.01–7.1)	*0.35*
Type D personality; vs. Non-Type D	6.00 (4.2–7.22) vs. 6.07 (4.22–7.49)	*0.8*	5.5 (2.9–7.1) vs. 6.06 (4.14–7.1)	*0.48*
Previous diagnosis of depression vs. None	6.87 (2.92–7.85) vs. 6.00 (4.22– 7.17)	*0.19*	5.61(3.00–6.90) vs. 5.74 (3.99–7.10)	*0.7*
BDI > 13 vs. ≤ 13	6.00 (4.14–7.65) vs. 6.15 (4.36–7.26)	*0.62*	5.01(2.62–7.37) vs. 5.99 (4.68–7.00)	*0.57*
BDI ≥ 29 vs. BDI <29	4.72 (4.30–7.49) vs. 6.20 (3.98–7.19)	*0.31*	4.27 (2.52–6.5) vs. 5.98 (4.01–7.13)	*0.08*
Anxiety vs. No anxiety (self report)	6.46(3.77–7.87) vs. 6.00 (4.22 –7.22)	*0.25*	5.01 (2.56–6.66) vs. 5.84(4.0–7.20)	*0.09*
Sex (male vs. female)	6.00 (4.2–7.2) vs. 6.36 (4.18–7.19)	*0.43*	5.59 (3.4–7.03) vs. 5.97 (4.75–7.6)	*0.26*
Age (greater than 70 vs. ≤70 years)	7.02 (6.6– 7.23) vs. 6.00 (4.1–7.46)	*0.21*	6.99 (5.94–7.00) vs. 5.61 (3.4–7.1)	*0.23*
ESS ≥ 10 vs. <10	6.02 (4.14–7.45) vs. 6.0 (4.63–7.73)	*0.24*	5.6 (3.4–7.1) vs. 6.1 (4.17–7.2)	*0.20*
4% desaturation index >20 vs. ≤20 per hour	6.0 (4.1–7.5) vs. 6.04 (4.16–7.38)	*0.8*	6.14 (3.62–7.27) vs. 5.39 (3.2–6.99)	*0.38*

**Table 3:** Distribution of subjects using CPAP for equal to/more than, or less than 4 h per night by study characteristics

	First follow-up (6–10 weeks)		6 month follow-up		OR for suboptimal compliance at six months (95% confidence interval)
	Percentage with characteristic and compliance ≤ 4 h/night	*P* value	Percentage with characteristic and compliance ≤ 4 h/night	*P* value	
Long term unemployed vs. people in work (or retired)	45% vs. 19%	*0.046*	64% vs. 26%	*0.011*	4.59 (1.4-15.0)
Education: Bachelor degree vs. less, formal education	22.2% vs. 12.9%	*0.18*	29.3% vs. 16.3%	*0.09*	1.12 (0.99-1.28)
Type D personality vs. non-Type D	21% vs. 19%	*0.40*	33% vs. 22%	*0.056*	1.30 (0.93-1.72)
Previous diagnosis of depression vs. None	27% vs. 18%	*0.088*	34% vs. 25%	*0.10*	1.34 (0.91-1.99)
BDI > 13 vs. ≤ 13	24% vs. 18%	*0.28*	34% vs. 21%	*0.03*	1.40 (1.03-1.9)
BDI ≥ 29 vs. BDI <29	29% vs. 19%	*0.24*	50% vs. 25%	*0.028*	2.65 (1.12-6.3)
Anxiety vs. No anxiety (self report)	30% vs. 19%	*0.052*	40% vs. 25%	*0.048*	1.67 (0.99-2.84)
Sex (male vs. female)	20.5% vs. 23.8%	*0.39*	30.2% vs. 20.5%	*0.14*	*1.68 (0.75-3.77)*
Age (greater than 70 vs. ≤70 years)	22.2% vs. 0%	*0.32*	29.9% vs. 0%	*0.19*	1.04 (1.0-1.07)
ESS ≥ 10 VS < 10	29% vs. 16%	*0.24*	29.3% vs. 19.2%	*0.20*	1.07 (0.96-1.18)
4% desaturation index >20 vs. ≤20 per hour	20% vs. 23%	*0 .34*	28% vs. 30%	*0.47*	1.04 (0.76-1.4)

**Fig. 3:**
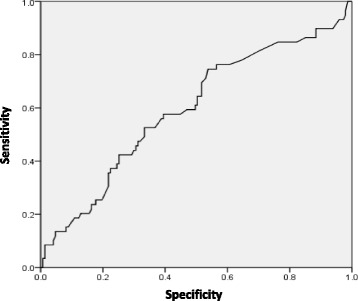
ROC analysis of physician’s assessment as a predictor of suboptimal compliance at 6 months

The diagnosing physicians in clinic who recommended the use of CPAP treatment correctly allocated higher and lower likelihoods of compliance to the good and poor complier populations. The group who used CPAP for ≥4 h/night at first visit, were given a likelihood of good compliance of 81%, while those who used CPAP < 4 h/night were given a likelihood of 71%. The difference persisted at 6 months. We looked for a specific cut off point for a physician’s initial impression to predict sub-optimal compliance. Though the sensitivity of predicting suboptimal compliance was 82.5% and specificity 91.1%, when a physician marked ≤ 60% on a visual analogue scale, on ROC analysis area under the curve was only 0.59 (Fig. 3).

At 6 months, the median compliance in individuals with an elevated BDI 2 score (>13) was 5.0 (IQR 2.6 – 7.4) hours/night which was non-significantly lower than the 6.0 (IQR 4.7 – 7.0) hours/night for those whose BDI score was ≤ 13 (Table 2). This difference became more marked for those with BDI score ≥ 29 and was statistically significant (*p* = 0.028). Current symptoms as measured by a raised BDI predicted poor compliance with CPAP; whereas an existing (and in many cases treated) diagnosis of depression did not. Depression was more common in the subjects who were unemployed and if they were excluded from the analysis there was no longer an association between poor compliance and depression. Self reported anxiety on enrolment to the study also predicted lower hours of use CPAP (Table 2). There was a non-significant trend for individuals with type D personality to comply sub-optimally at 6 months, compared to those who were non-type D. No association was found between hours of use either at first or 6 month follow up and sex, age, baseline ESS, or ODI (Table 2).

## Discussion

CPAP treatment for Obstructive sleep apnoea syndrome is rapidly effective with sleepiness and other symptoms best controlled in those who use CPAP for the highest number of hours [6, 8, 9, 26]. It will be best clinical practice and use of resources to identify patients who are likely to have sub-optimal compliance and concentrate interventions to improve usage on those people. In the current study SEC for those in work, level of education and personality type, the severity of sleep apnoea measured by ESS or by ODI, were not good predictors. Our study has shown that the Gestalt impression of the prescribing physician can predict to an extent which patients will be good and less good compliers. Less operator dependent measures, which differentiated these patients at presentation were employment status and a diagnosis of depression.

To date there have been few studies looking at the role of socio-economic status in acceptance and compliance with CPAP treatment in patients with OSAHS. One cross sectional study looking at the pattern of adherence and acceptance of CPAP in an Israeli population found the likelihood of CPAP purchase to be determined by income level [19]. Only 21.8% of individuals with low income accepted and purchased CPAP compared to 51.4% from average and 75.6% from higher income groups. Twenty nine percent of those who declined CPAP cited cost as the reason. In our study no difference in the average compliance or the proportion of people using CPAP sub-optimally, was seen in relation to different socio economic class for those in work. CPAP was provided free of charge at the point of delivery making income level unlikely to be a determinant. There was however significantly lower compliance in individuals who were long term unemployed compared to the ones who were in work with an odds ratio of 4.6 for sub-optimal compliance at 6 months. The number of unemployed individuals was small (19 at the start of the study), and this result may not be generalizable. However, the number of long term unemployed people in the study reflected the unemployment rate in the UK at the time of the study. A study done in a health care system similar to ours found no difference in the compliance between individuals depending on socio-economic background assessed by educational status [27].

Individuals who have high negative affectivity and social inhibition, are classed as Type D personality [25]. Negative emotions in these individuals are likely to be associated with self-neglect with possible implications of poor adherence to treatment. A retrospective study previously described that compliance in those with Type D personality was on an average 1 h 20 min less than those without. Also only 50% of individuals used CPAP ≥4 h/night, compared to 84% of the non-type D personality [22]. Prospectively, in our study individuals with type D personality showed no difference in median compliance compared with non-type D. There was a non-significant trend towards suboptimal compliance with 33% of individuals with type D personality complying sub-optimally compared to 22% with non-type D. It is not clear as to why our results were different than to those of the previous study. A likely explanation is that previous researchers looked at the association retrospectively leading to a recall bias. It is also conceivable that some of the responses to assess social inhibition and negative affectivity may have altered over a period of time, particularly with CPAP intervention that potentially can influence mood and behaviour. This may have led to a smaller proportion of patients with type D in the retrospective study (30% vs 45%) and hence the differences in compliance.

The prevalence of depression as diagnosed by the GP’s, in our population with OSAS offered CPAP, was 32.7% which is higher than estimates for otherwise unselected primary care patients but similar levels found by other investigators (32-48%) in people with OSAS [28, 29]. In our study, current symptoms of depression as measured by a high BDI-2 scores predicted lower compliance at 6 months. Those with a diagnosis of anxiety were less likely to comply optimally at first visit and 6 months. Depression is known to be associated with poor adherence to therapy in other chronic medical disorders [30, 31]. In patients with OSA there is conflicting evidence regarding any association between underlying depression and CPAP use. In a prospective study involving 70 patients, there was no correlation between CPAP compliance at 1 month and underlying depression measured by the Hospital Anxiety and Depression Scale (HADS) scores [32]. In another study using the Minnesota Multiphasic Personality Inventory to measure depression, individuals who were depressed had poor CPAP compliance compared to those who were not depressed [33]. A more recent study found that depression measured on HADS predicted lower Auto CPAP use at one week (R^2^ = 0.19, *p* <0.001) [34]. Unemployed individuals, compared to those in work, have a greater chance of poor adherence to chronic medical therapy [35]. They are also more likely to have clinical depression than employed individuals [36]. However, cause and effect association is not clearly established. In our study it is difficult to separate out the effects of depression and unemployment which frequently occurred in the same individuals.

To the best of our knowledge this is the first study to investigate the confidence of a physician in predicting compliance, when they reviewed the patient for the first time. There was an association between a physician’s confidence that the individual would use CPAP and whether they used it 4 or more hours/night. The differences observed were not big; however, they support the hypothesis that a physician can predict CPAP compliance when initiating the treatment. We did not find any association between the physiological variables of severity of OSA, ESS score and CPAP compliance, which has been the experience of other investigators [13, 37, 38].

There are some limitations to the study to consider. The sample size was determined to give adequate power primarily for the SEC status comparisons. There are a number of non-significant trends in the results which might have been found by chance or could have been significant in a larger study. The treatment was free for our subjects but in many health care systems this will not be the case and income levels may be a dominant factor. This affects the generalisability of our results. More than a third of patients seen in clinic did not consent to participation in the study. This may have introduced a selection bias, recruiting individuals with a higher rate of acceptance and compliance but previous audits of our service have shown very similar rates for these parameters [39]. The clinicians involved in scoring the likelihood that subjects would comply with CPAP had a large range of experience. It is possible that if we had focussed just on more experienced staff that the predictive power of the clinician’s impression would have been greater. This might form the basis of a further study.

In this cohort overall initial acceptance of CPAP was 89.8% which compares favourably with published data. However, as in other studies a proportion of patients do not comply well with CPAP. As in other studies early levels of compliance measured as hours of use was highly correlated with long term use (at 6 months). The doctors prescribing CPAP gave patients who complied poorly a higher chance of not complying when asked to predict their usage. People who were long term unemployed and those with a high score on BDI-2 were less likely to use CPAP for ≥4 h/night. Socio-economic status, education level, ESS and ODI did not predict good and poor compliers.

## Conclusions

In our practice there is no significant association between CPAP compliance with socio-economic status, education or personality type. Long term unemployed or depressed individuals were less likely to use CPAP optimally and may need more intensive support to gain the optimal benefit from CPAP. Our findings suggest that people who are long term unemployed and/or currently depressed should be identified and offered more intensive support when initiating CPAP therapy to give them the best chance of getting on with the treatment and achieving the benefits that it can offer.

## Abbreviations

AHI: Apnoea Hypopnoea Index
BDI-2: Beck’s depression inventory 2
BMI: Body mass index
CPAP: Continuous positive airway pressure
HAD: Hospital anxiety and depression scale
OSA: Obstructive sleep apnoea
SEC: Socio economic class
